# The *Journal of Therapeutic Ultrasound*: broadening knowledge in a rapidly growing field

**DOI:** 10.1186/2050-5736-1-1

**Published:** 2013-04-25

**Authors:** Robert Muratore, Arik Hananel

**Affiliations:** 1 Quantum Now LLC, Huntington, NY, USA; 2 Focused Ultrasound Foundation, Charlottesville, VA, USA; 3 Department of Radiation Oncology, University of Virginia, Charlottesville, VA, USA

## Abstract

The Journal of Therapeutic Ultrasound has been established to provide an open access, online venue for the exponentially growing body of work in biomedical ultrasound therapy.

## 

Welcome to the *Journal of Therapeutic Ultrasound* (JTU), a newly launched, online, open-access journal designed to accelerate the development of focused ultrasound and its adoption in the clinic.

Biomedical therapy was among the earliest applications of ultrasound, preceding solid state electronics and inexpensive computers by decades. However, there was insufficient commercialization for therapeutic ultrasound devices to be grandfathered into the Medical Device Amendments of 1976 to the United States Federal Food, Drug, and Cosmetic Act [1]. Therefore, regulatory approval is required for new therapeutic ultrasound devices in the United States. Similar regulatory burdens exist worldwide. The first such device to be approved in the United States was the Sonocare CST-100 (Sonocare, Inc., Upper Saddle River, NJ, USA), designed for the treatment of glaucoma and approved in 1988 [2]. In the year that the first patent covering the Sonocare system was filed, 1980 [3], only about 16 articles were published with the phrase ‘therapeutic ultrasound’ in the title. Since then, the annual number of publications in therapeutic ultrasound has been growing exponentially, reaching approximately 1,440 articles in 2011. Figure 1 demonstrates the exponential fit: the number of articles *n* = 2 *exp*((*t* - 1970)/6), with coefficient of determination *R*^2^ = 0.965 and time constant *τ* = 6 years. In 2011, therapeutic ultrasound received attention from the popular press; following a presentation at TEDMED 2011 by Dr. Yoav Medan [4], *Time Magazine* named focused ultrasound therapy as one of the 50 Best Inventions of 2011 in its 2011 November 28 issue [5].

**Figure 1:**
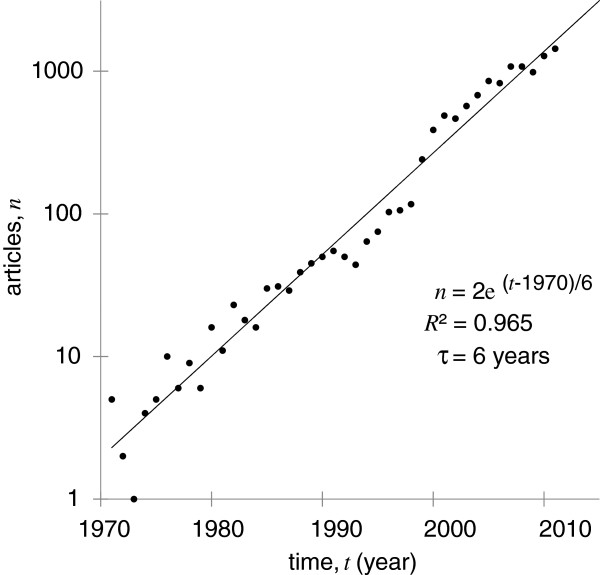
**A *Google Scholar *search for the title phrase ‘therapeutic ultrasound’. **Restricting the search by year provides an estimate for annual publishing activity. A fitted exponential model predicts 3,600 articles in 2015.

Adoption of new International Electrotechnical Commission standards (e.g., SC62D Project Team 60601‐2‐62) by regulatory agencies such as the United States Food and Drug Administration (FDA), the European Commission, the Japanese Pharmaceuticals and Medical Device Agency (PMDA), and the Chinese State Food and Drug Administration (SFDA) will enable more rapid approval of devices and, therefore, more clinical applications. Thus, the exponential growth is likely to continue with a predicted output of 3,600 articles by 2015.

In order to serve this growing demand, this journal was originally proposed by Dr. Neal Kassell, Chairman of the Focused Ultrasound Foundation, in collaboration with Dr. Lawrence Crum, Past President of the International Society for Therapeutic Ultrasound (ISTU). JTU serves as the official journal of these two organizations. The 26 members of its editorial board represent 10 countries.

The Journal accepts research articles, case reports, reviews, meeting reports, and study protocols, along with supplemental materials. Appropriate topics include translational and clinical research in all areas of therapeutic ultrasound, including stimulation, inhibition, destruction, or modification of tissue function or structure via focused ultrasound.

Peer review follows the traditional closed model with a minimum of two reviewers. Authors are encouraged to carefully follow reporting standards for ultrasound dose [6] and to follow the Journal's lead in adhering to guidelines of the Committee on Publication Ethics (COPE) [7] and the World Association of Medical Editors [8].

As an online journal, JTU offers authors unlimited color images and support for additional materials such as audio and video files. Another advantage is rapid publication following acceptance after successful completion of peer review.

Open access means that authors retain full rights to their articles through the Creative Commons license and that articles will reach a wide audience including developing countries and mainstream media. Articles will be deposited with PubMed Central upon publication without the 6- to 12-month delay which is common among restricted access journals.

Author fees are waived through 2014 through support provided by the Focused Ultrasound Foundation.

The Journal is intended to reach scientists and clinical practitioners interested in broadening their knowledge in the field of therapeutic ultrasound, including MR-guided and ultrasound-guided focused ultrasound surgery, high intensity focused ultrasound, and non-ablative ultrasound therapies. By reaching audiences around the world, it will play a key role in advancing clinical applications of therapeutic ultrasound.
